# Dietary Thymol–Carvacrol Cocrystal Supplementation Improves Growth Performance, Antioxidant Status, and Intestinal Health in Broiler Chickens

**DOI:** 10.3390/antiox14111323

**Published:** 2025-11-01

**Authors:** Jingzhe Yang, Changjin Li, Shuzhen Jiang, Yuemeng Fu, Guohui Zhou, Yufei Gao, Weiren Yang, Yang Li

**Affiliations:** Key Laboratory of Efficient Utilization of Non-Grain Feed Resources (Co-Construction by Ministry and Province), Ministry of Agriculture and Rural Affairs, Shandong Provincial Key Laboratory of Animal Nutrition and Efficient Feeding, College of Animal Science and Technology, Shandong Agricultural University, Panhe Street 7, Tai’an 271017, China; yangjingzhe0306@126.com (J.Y.); 17664393914@163.com (C.L.); szjiang@sdau.edu.cn (S.J.); fuyuemeng@yeah.net (Y.F.); zhou_guohui@yeah.net (G.Z.); 15676229908@163.com (Y.G.); wryang@sdau.edu.cn (W.Y.)

**Keywords:** antioxidation, broiler, essential oil, intestinal health, oxidative stress, performance

## Abstract

This study investigated the impacts of dietary thymol–carvacrol cocrystal (CEO) supplementation on broiler production performance, antioxidant status, intestinal health, and cecal microbiota. Eight hundred one-day-old chicks were randomly divided into four groups, receiving basal diets supplemented with 0, 40, 60, or 80 mg/kg CEO. The results showed that CEO addition increased average daily gain, superoxide dismutase activity in the serum, liver, and jejunum, jejunal villus height/crypt depth ratio, cecal butyric acid concentration, and Lactobacillus abundance, while reducing serum alanine transaminase activity and malondialdehyde content in the serum, liver, and jejunum. Furthermore, 60 mg/kg CEO enhanced the final body weight, dressing percentage, serum total protein and glucose levels, and jejunal trypsin and amylase activities, while lowering the feed-to-gain ratio and serum cholesterol, urea nitrogen, and aspartate transaminase concentrations; it also increased the activities of superoxide dismutase, catalase, and glutathione and mRNA expressions of related genes in the liver and jejunum. It also increased cecal concentrations of acetic acid and isovalerate acid, while decreasing serum diamine oxidase and D-lactate concentrations, as well as malondialdehyde concentrations in the serum, liver, and jejunum. Therefore, dietary CEO supplementation improved the production performance, antioxidant status, and liver and gut health and function in broilers, with 60 mg/kg CEO demonstrating the most pronounced effects.

## 1. Introduction

In modern intensive poultry production, broilers are often exposed to various stressors, including high stocking densities, environmental fluctuations, and suboptimal management practices, which can result in oxidative stress [1]. Oxidative stress occurs when an imbalance in redox homeostasis leads to an excessive reactive oxygen species (ROS) generation, leading to proteins, lipids, and/or DNA damage. Broiler health and farm profitability are increasingly compromised by this growing challenge in commercial poultry production [2]. To mitigate these negative effects caused by oxidative stress, the use of natural antioxidants has gained significant attention in recent years.

Essential oils (EOs), derived from plant extracts, have emerged as promising alternatives in poultry production due to their potent antioxidant properties, along with being green, safe, and non-toxic [3]. Thymol and carvacrol, the two primary bioactive constituents of commonly used herbs such as oregano and thyme, exhibited potent antioxidant, anti-inflammatory, and antibacterial activities when applied in poultry production [4]. Remarkably, thymol and carvacrol displayed greater antioxidant efficacy when combined than when applied alone, highlighting their synergistic interaction [5]. Hashemipour et al. [6] showed that adding 60, 100, and 200 mg/kg thymol and carvacrol (1:1; 50% active components) could linearly increase the BW gain and feed efficiency and enhance the antioxidant and digestive enzyme activities and immune function of broilers. Ding et al. [7] reported that broiler diets with essential oils containing thymol and carvacrol (1:2) at doses of 200, 400, or 600 mg/kg enhanced the ADG and feed conversion ratio over a 48 d trial period, with the 400 mg/kg supplementation yielding the most pronounced beneficial effects. However, thymol and carvacrol are highly volatile, prone to oxidation, and poorly soluble in water, significantly compromising their stability, bioavailability, and biological efficacy [8]. These limitations hinder their widespread adoption in the poultry industry.

Cocrystal technology represents a cutting-edge method in drug formulation, significantly improving critical pharmaceutical properties such as solubility, stability, and bioavailability [9]. The physicochemical properties and bioactivity of thymol and carvacrol have been proven to be greatly enhanced by this novel approach [10]. A recent study in broilers indicated that supplementation with only 30 mg/kg thymol–carvacrol cocrystals (CEO), equivalent to a 50% active dose, not only improved the growth performance but also promoted intestinal health through enhancing anti-inflammatory and antioxidant capacities [11]. Our previous study demonstrated that a dietary inclusion of 60 mg/kg CEO, which had an effective content of 25%, boosted antioxidant capacity, thereby improving meat quality and nutritional value in broilers [3]. This seemed to suggest that the CEO improved broiler production performance even at lower supplementation levels, compared with the direct addition of individual thymol and carvacrol. Despite the promising advantages of CEO, current research on its application effects in broilers remains insufficient, and the optimal dosage for its application in broilers requires further investigation.

Based on the above information, the current study was aimed to assess the efficacy of CEO in improving production performance, antioxidant capacity, intestinal development and function, and cecal microbiota composition, clarifying the optimal dosage of CEO in the feed for broiler chickens. The findings of this research could pave the way for the development of innovative, natural feed additives that enhance broiler health and productivity while reducing the reliance on synthetic antioxidants and antibiotics.

## 2. Materials and Methods

### 2.1. Animals and Treatments

A total of 800 one-day-old male Arbor Acres chicks, each with a mean BW of 45.02 ± 0.03 g, were randomly divided into four groups in a completely randomized design, with each group comprising eight replicates and each replicate consisting of 25 chicks housed per cage over a 42 day experimental period. The broilers were administered a basal diet supplemented with varying concentrations of CEO at 0, 40, 60, and 80 mg/kg, corresponding to the groups CEO0 (control), CEO40, CEO60, and CEO80, respectively. The CEO product, sourced from Cocrystal Health Co., Ltd. (Jiashan, China), contained an effective concentration of 25% thymol and carvacrol in a 1:1 ratio, as detailed by [3]. The composition of the basal diets (Supplemental Table S1) was formulated in accordance with the nutritional standards set forth by [12] and the Aviagen Company [13] for broiler chickens, utilizing a three-phase feeding regimen (starter phase: days 1–14; growth phase: days 15–28; finishing phase: days 29–42). Broilers were housed in a temperature-controlled environment with a specified lighting schedule (24 h on day 1, 23 h from days 2 to 7, and 20 h from days 8 to 42). They were kept in a three-tiered metal coop (120 × 70 × 40 cm), defined as one cage, which served as the experimental unit for the study. Following a first week at 35 °C, the room temperature was decreased daily by 0.5 °C until stabilizing at 23 °C [14]. The relative humidity was maintained at 60 ± 0.8% for the first week and then at 50 ± 0.8% for the subsequent five weeks. All the broilers were provided with ad libitum access to water and feed.

### 2.2. Sampling

On day 42 of the trial, a total of 32 broilers (one from each replicate, with BWs close to the replicate average) were selected for sample collection after a 12 h feed withdrawal period. The live weight of individual birds was recorded before sampling. Around 5 mL of a blood sample was collected via the wing vein into the coagulation-promoting tubes containing heparin sodium. The serum sample was harvested, after being centrifuged at 4000 rpm for 15 min, and stored at −35 °C. After the broiler was euthanized via cervical distension and dislocation, the chest of the broiler was opened, and the organs (liver, bursa of Fabricius, spleen, thymus, glandular stomach, muscular stomach, small intestine, and large intestine), as well as abdominal adipose, leg muscle, and breast muscle, were removed and weighed. A tissue sample was excised from the left lobe of the liver and rinsed with an ice-cold saline solution. Subsequently, the liver tissue was immediately frozen with liquid nitrogen after being surface-dried with filter paper and stored at −80 °C. Also, a segment approximately 10 cm in length was taken from the middle portion of the small intestine. Approximately 2 cm of the sample was preserved in a 4% paraformaldehyde (PF) solution for 24 h at room temperature. The remaining intestinal segment was rinsed with saline solution, after which the mucosa was scraped and stored in a 2 mL cryovial. The mucosa samples underwent rapid freezing using liquid nitrogen and were subsequently stored at −80 °C. Furthermore, the jejunal and cecal contents were collected into sterile bags and stored at −80 °C for subsequent analyses of digestive enzyme activities and bacterial DNA extraction, respectively.

### 2.3. Determination of Growth Performance

Feed consumption was recorded daily for each cage throughout the experimental period, with these measurements serving as the basis for calculating the average daily feed intake (ADFI). At days 21 and 42 of the trial, all birds were weighed on a per-cage basis after a 12 h fasting period to compute the average daily gain (ADG). The ratio of feed to gain (F/G) was determined by dividing the ADFI by the ADG. Additionally, the survival rate was calculated by dividing the final number of broilers by the initial number, excluding all mortalities and culls.

### 2.4. Determination of Slaughter Performance

The semi-eviscerated weight and full eviscerated weight were measured, and the weights of abdominal fat, leg muscle, and breast muscle were recorded according to previous methods [15]. The percentages of semi-eviscerated yield, full eviscerated yield, abdominal fat, leg muscle, and breast muscle, along with the dressing percentage, were calculated.

### 2.5. Serum Biochemical Parameters Measurement

The blood biochemical parameters, including total protein (TP, CAS#A045-4-2), triacylglycerol (TG, CAS#A110-1-1), total cholesterol (TC, CAS#A111-1-1), glucose (GLU, CAS#A154-1-1), urea nitrogen (UREA, CAS#C013-3-1), high-density lipoprotein (HDL, CAS#A112-1-1), low-density lipoprotein (LDL, CAS#A113-1-1), alkaline phosphatase (ALP, CAS#A059-2-2), aspartate transaminase (AST, CAS#C010-2-1), and alanine transaminase (ALT, CAS#C009-2-1), were measured on a Roche automated biochemical analyzer (Roche Diagnostic System Inc., Zurich, Switzerland) using the commercial kits (Jiancheng Bioengineering Institute, Nanjing, China).

### 2.6. Measurement of Antioxidant Parameters

The activities of antioxidant enzymes, including superoxide dismutase (SOD, CAS#A001-3-2), catalase (CAT, CAS#A007-1-1), and glutathione peroxidase (GSH-Px, CAS#A005-1-2), and reduced glutathione (GSH, CAS#A006-2-1) and malondialdehyde (MDA, CAS#A003-1-2) concentrations in the serum, liver, and small intestine were determined using the methods in previous studies [16,17]. The SOD activity was quantified using the WST-1 method, which employs a xanthine/xanthine oxidase system to generate superoxide anions (O_2_^∙−^). The WST-1 compound forms a water-soluble formazan dye upon reduction by O_2_^∙−^, and the inhibition of this reduction process was used to determine SOD activity at 450 nm. The activity of CAT was assessed by monitoring the rate of hydrogen peroxide (H_2_O_2_) decomposition at a wavelength of 240 nm. The activity of GSH-Px was calculated by measuring the rate at which it catalyzed the reaction between glutathione and H_2_O_2_ at 412 nm. The GSH content was determined colorimetrically at 405 nm based on its reaction with 5,5′-dithiobis-(2-nitrobenzoic acid), which yields a yellow-colored product. MDA concentration was determined through its reaction with thiobarbituric acid at 95 °C, resulting in red-colored compounds with an absorption peak at 532 nm. The commercial kits were purchased from Jiancheng Bioengineering Institute (Nanjing, China).

### 2.7. Serum Diamine Oxidase (DAO) and D-Lactate Concentrations Determination

The serum concentrations of DAO (CAS#A088-3-1) and D-lactate (CAS#A019-3-1) were determined using the ELISA kits (Jiancheng Bioengineering Institute) on the grounds of the manufacturer’s protocol [18]. All samples were analyzed in duplicate.

### 2.8. Intestinal Morphological Measurement

The fixed intestinal segments were removed from 4% PF solution, dehydrated with ethanol and xylene, and embedded in paraffin according to conventional histological methods [19]. Serial 5 μm sections were cut using a Leica RM2135 microtome (Wetzlar, Germany) and routinely stained with H&E. Photomicrographs were captured at 100× magnification with an Eclipse 80i microscope (Nikon, Tokyo, Japan). Crypt depth (CD) and villus height (VH) were measured using the JD801 morphologic analysis system (JEDA, Nanjing, China), and the VH to CD (V/C) ratio was computed, as previously described [17].

### 2.9. Determination of Digestive Enzyme Activities

The jejunal chyme samples were homogenized using frozen phosphate-buffered saline (PBS; pH 7.0) at a 1:9 ratio, and centrifuged at 3000 rpm for 10 min. Then, the supernatants were collected for measurements of the trypsin, lipase, and amylase activities using the reagent kits from Nanjing Jiancheng Bioengineering Institute, following the manufacturer’s protocols.

### 2.10. Determination of Gene Expression in Liver and Small Intestine

About 0.1 g frozen intestinal mucosa and liver tissue samples were ground into powder with liquid nitrogen in a pre-cooled mortar. Total RNA was isolated from the tissues using TRIzon reagent (Invitrogen, Carlsbad, CA, USA), followed by the measurement of RNA concentration with a NanoDrop2000 Spectrophotometer (Thermo Scientific, Lenexa, KS, USA) and assessment of RNA integrity through agarose gel electrophoresis. The procedures of reverse transcription of RNA and real-time quantitative PCR were conducted according to the methods described by Liu et al. [20]. The PCR amplification reaction was prepared in a 10 µL volume consisting of 4.0 µL of cDNA, 5 µL of 2 × SYBR^®^ Green Pro Taq HS Premix, 0.4 μL ddH_2_O, and 0.3 µL of each primer. The primers (Supplemental Table S2) were designed and synthesized by Accurate Biology. The amplification conditions were as follows: 35 cycles of 95 °C for 15 s, 60 °C for 15 s, and 72 °C for 15 s. β-actin was used as an internal control and amplified in parallel with the target genes for normalization, and gene expression quantification was performed using the 2^−ΔΔCt^ method. Each sample was performed in triplicate.

### 2.11. Determination of Cecal Short-Chain Fatty Acid (SCFA) Concentrations

Approximately 50 mg of cecal content was used to measure the concentrations of cecal SCFAs (acetic acid, propionic acid, isobutyric acid, butyric acid, valeric acid, isovalerate acid, and caproate acid) through ultra-performance liquid chromatography/tandem mass spectrometry (UPLC/MS/MS), as previously described [21]. A Waters ACQUITY UPLC I-Class system (Waters, Milford, MA, USA) coupled to a triple quadrupole mass spectrometer (Xevo TQ-S, Waters, Milford, MA, USA) was used for the analysis at Hexin Technology Co., Ltd. (Beijing, China) [11].

### 2.12. Cecal Microbial Composition and Diversity Analysis

Six cecal content samples from each group were selected for microbial analysis. Bacterial genomic DNA from cecal contents was extracted with an E.Z.N.A.™ Stool DNA Kit (Omega Bio-Tek, Norcross, GA, USA), followed by DNA concentration quantification and purity assessment [22]. The V4 region of 16S rDNA was amplified with 515 F/806R primers, with sequencing using Illumina NovaSeq 6000 platform (Novogene, Beijing, China). The generated 250 bp paired-end sequencing reads were merged using the FLASH tool [23] and processed with QIIME2 for quality control and feature table creation [7]. Sequences with 97% similarity were grouped into operational taxonomic units (OTUs) using UPARSE and identified to various taxonomic levels with the SILVA database [7,22]. Chao 1, Shannon, Simpson, Dominance, Pielou, and Observed_feature indexes were selected to assess variations in alpha diversity across different groups. Beta diversity based on Bray–Curtis distances was computed and visualized through Principal Coordinate Analysis (PCoA) and displayed in R software (version 4.1.1).

### 2.13. Statistical Analysis

The average cage data were employed to evaluate the impacts on growth performance, while individual broilers were treated as the experimental unit for the analysis of other variables. The Shapiro–Wilk test (W > 0.05) was used for data normality assessment. The PROC GLIMMIX procedure of SAS (version 9.4; SAS Institute, Cary, NC, USA) was employed for analyzing the abundance of cecal microbiota, and significant differences among the microbial communities were assessed using the analysis of similarity (ANOSIM) test. Other data were statistically analyzed using the PROC GLM procedure in SAS. Tukey’s multiple comparison test was applied for the multiple comparisons of treatment means. Polynomial contrasts were used to assess the linear and quadratic effects of increasing levels of dietary CEO addition. Results are presented as mean ± SE in figures and as means with the SEM in tables. *p* < 0.05 was considered statistically significant.

## 3. Results

### 3.1. Growth Performance and Slaughter Performance

As shown in Table 1, the BWs of broilers in the CEO60 group were the highest among the four groups on days 21 and 42 of the trial, and were higher than those in the other three groups (*p* < 0.05). On day 21, the BWs in the CEO40 and CEO80 groups were also greater than the BW in the control group (*p* < 0.05). Additionally, broilers in the CEO60 group exhibited the highest ADG across all periods: days 0 to 21, 22 to 42, and 0 to 42. Specifically, the ADG in the CEO60 group was higher than the ADG in the control, CEO40, and CEO80 groups from day 0 to 21 (*p* < 0.05), higher than that in the control group from day 22 to 42 (*p* < 0.05), and higher than that in the control and CEO40 groups from day 0 to 42 (*p* < 0.05). Furthermore, the ADG in the control group was lower than those in the CEO40 and CEO80 groups from day 0 to 21 and 0 to 42 (*p* < 0.05). Regarding the ADFI, the CEO80 group showed higher than the other three groups from day 0 to 21 (*p* < 0.05), and higher than the control group from day 0 to 42 (*p* < 0.05). The F/G ratio was markedly lower in the CEO40 and CEO60 groups relative to the control and CEO80 groups from day 0 to 21 (*p* < 0.05). Moreover, the CEO60 group exhibited a lower F/G ratio than the control group from day 22 to 42 (*p* < 0.05), as well as compared to the control and CEO80 groups from day 0 to 42 (*p* < 0.05). In addition, the BWs on days 21 and 42, the ADGs across all periods, and the ADFIs from day 0 to 21 and 0 to 42 showed linear (*p* < 0.05) and quadratic (*p* < 0.05) increases with increasing dietary CEO supplementation; the F/Gs across all periods displayed quadratic changes (*p* < 0.05) as dietary CEO addition increased. The survival rates were higher in the CEO40 and CEO60 groups than in the control group (*p* < 0.05), with the CEO60 group also higher than the CEO80 group (*p* < 0.05). Overall, the survival rate demonstrated a significant quadratic dose–response relationship to increasing dietary CEO levels (*p* = 0.010). Additionally, increasing CEO addition quadratically changed the dressing percentage of broilers (*p* < 0.05), and the CEO60 group had a higher dressing percentage compared with the control group (*p* < 0.05). The percentages of semi-eviscerated yield, full eviscerated yield, abdominal fat, leg muscle, and breast muscle showed no significant differences among the four groups (*p* > 0.05; Supplemental Table S3).

### 3.2. Serum Biochemical Parameters

The data of serum biochemical parameters in broilers are displayed in Table 2. The TP concentration was higher in the CEO60 group than in the other three groups (*p* < 0.05). Relative to the control group, the CEO60 group reduced TG and UREA concentrations (*p* < 0.05), and increased GLU concentration (*p* < 0.05) in the serum. Serum TG (quadratic, *p* = 0.031), GLU (quadratic, *p* = 0.032), and UREA (linear, *p* = 0.010; quadratic, *p* = 0.015) concentrations exhibited dose-dependent changes in response to increasing CEO supplementation. Moreover, the dietary addition of CEO decreased serum ALT concentration relative to the control group (*p* < 0.05), and the serum ALT concentration of the CEO60 group was also markedly lower than that of CEO40 group (*p* < 0.05). In addition, 60 and 80 mg/kg CEO supplementation reduced serum AST activity compared with the control group (*p* < 0.05). The serum ALT and AST concentrations showed linear (*p* < 0.05) and quadratic (*p* < 0.05) decreases with increasing CEO addition.

### 3.3. Serum Diamine Oxidase and D-Lactate Concentrations

As shown in Table 3, the serum DAO activity was lower in the CEO60 group than in the other three groups (*p* < 0.05), and, compared to the control and CEO40 groups, the CEO60 and CEO80 groups demonstrated lower serum D-lactate concentrations in the broilers (*p* < 0.05). Both the DAO and D-lactate concentrations displayed linear (*p* < 0.05) and quadratic (*p* < 0.05) decreases as CEO supplementation increases.

### 3.4. Antioxidant Parameters in the Serum, Liver, and Small Intestine

The effects of dietary CEO addition on the antioxidant parameters in the serum, liver, and small intestine are shown in Table 4. Supplementation with CEO, particularly at 60 mg/kg, improved antioxidant parameters across tissues. The CEO60 group had elevated activities of SOD, CAT, GSH-Px, and GSH in the serum (*p* < 0.05), SOD, CAT, and GSH in the liver and small intestine (*p* < 0.05), and concurrently reduced MDA levels in all three tissues (*p* < 0.05) compared to the control. The regression analysis revealed that SOD and CAT showed linear and/or quadratic increases (*p* < 0.05), while MDA content decreased (*p* < 0.05) linearly and quadratically in the serum, liver, and small intestine. Serum GSH-Px and GSH, as well as hepatic GSH, were also enhanced (linear and/or quadratic, *p* < 0.05) by increasing CEO additions.

### 3.5. Small Intestinal Morphological Measurements

**Figure 1:**
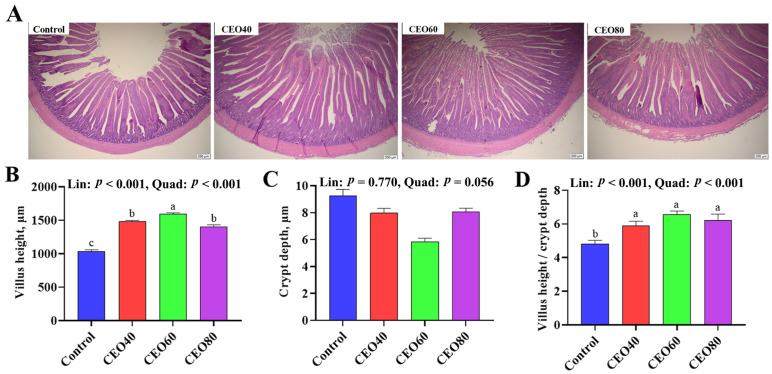
Effects of thymol–carvacrol cocrystals on intestinal morphology of broilers at 42 days of age. (**A**) Small intestinal section stained with hematoxylin–eosin (scale bar = 200 μm); (**B**) villus height; (**C**) crypt depth; (**D**) villus height/crypt depth. Control: basal diet without thymol–carvacrol cocrystal supplementation. CEO40, CEO60, and CEO80: basal diet supplemented with thymol–carvacrol cocrystals at 40, 60, and 80 mg/kg, respectively. Data in the bar chart are presented as mean ± SE. *n* = 8. a, b, c: means differ significantly (*p* < 0.05). Lin and Quad show linear and quadratic effects of different dietary CEO levels.

As displayed in Figure 1, the dietary inclusion of CEO increased the small intestinal VH and V/C ratio compared to the control group (*p* < 0.05), and the VH was also greater in the CEO60 group than in the CEO40 and CEO80 groups (*p* < 0.05). Moreover, as dietary CEO addition increased, the intestinal VH and V/C ratio displayed linear (*p* < 0.001) and quadratic (*p* < 0.001) increases.

### 3.6. Small Intestinal Digestive Enzyme Activities Measurements

As shown in Table 5, the activities of trypsin and amylase were higher in the CEO60 group than those in the control groups (*p* < 0.05). The lipase activity did not differ among the four groups (*p* > 0.05). Increasing CEO additions enhanced trypsin (linear, *p* = 0.020; quadratic, *p* < 0.001) and lipase (linear, *p* = 0.020) activities, and quadratically changed (*p* = 0.023) amylase activity in the small intestine.

### 3.7. Antioxidant-Related Gene Expressions in the Liver and Small Intestine

#### 3.7.1. Gene Expressions in the Liver

**Figure 2:**
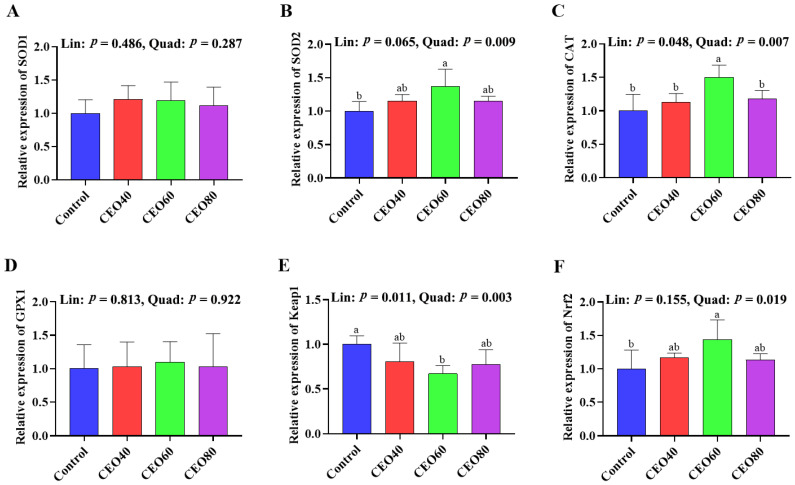
Effects of thymol–carvacrol cocrystals on the relative expression of antioxidant-related genes in the liver of broilers at 42 days of age. (**A**) *SOD1*, superoxide dismutase 1; (**B**) *SOD2*, superoxide dismutase 2; (**C**) *CAT*, catalase; (**D**) *GPX1*, glutathione peroxidase 1; (**E**) *Keap1*, kelch-like ECH-associated protein 1; (**F**) *Nrf2*, nuclear factor erythroid 2-related factor 2. Control: basal diet without thymol–carvacrol cocrystal supplementation. CEO40, CEO60, and CEO80: basal diet supplemented with thymol–carvacrol cocrystals at 40, 60, and 80 mg/kg, respectively. Data in the bar chart are presented as mean ± SE. *n* = 8. a, b: means differ significantly (*p* < 0.05). Lin and Quad show linear and quadratic effects of different dietary CEO levels.

As shown in Figure 2, compared to the control group, the *SOD2* and *Nrf2* mRNA expressions were up-regulated (*p* < 0.05), and the *Keap1* mRNA expressions were down-regulated in the CEO60 group (*p* < 0.05). The CEO60 group also showed a higher mRNA expression of *CAT* compared to the other three groups (*p* < 0.05). The *CAT* expression increased (linear, *p* = 0.048; quadratic, *p* = 0.007) and *Keap1* expression decreased (linear, *p* = 0.011; quadratic, *p* = 0.003) with dietary CEO addition increasing, and increasing dietary CEO additions quadratically changed (*p* < 0.05) the mRNA expressions of *SOD2* and *Nrf2*.

#### 3.7.2. Gene Expressions in the Small Intestine

**Figure 3:**
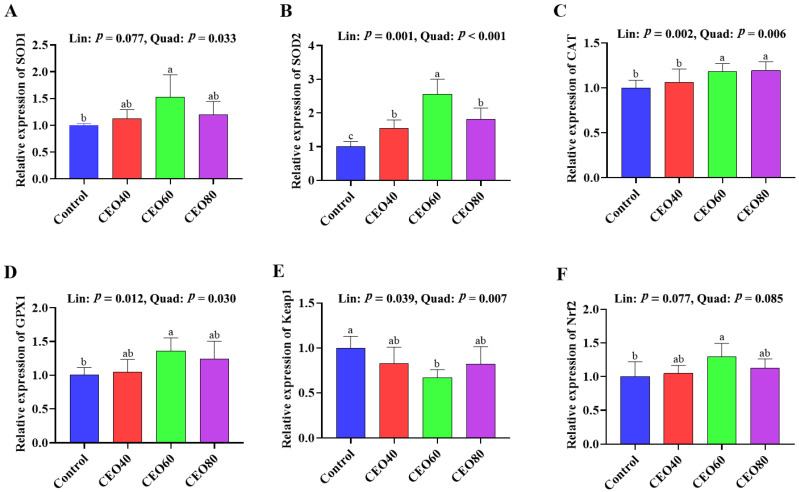
Effects of thymol–carvacrol cocrystals on the relative expression antioxidant-related genes in the small intestine of broilers at 42 days of age. (**A**) *SOD1*, superoxide dismutase 1; (**B**) *SOD2*, superoxide dismutase 2; (**C**) *CAT*, catalase; (**D**) *GPX1*, glutathione peroxidase 1; (**E**) *Keap1*, kelch-like ECH-associated protein 1; (**F**) *Nrf2*, nuclear factor erythroid 2-related factor 2. Control: basal diet without thymol–carvacrol cocrystals supplementation. CEO40, CEO60, and CEO80: basal diet supplemented with thymol–carvacrol cocrystals at 40, 60, and 80 mg/kg, respectively. Data in the bar chart are presented as mean ± SE. *n* = 8. a, b, c: means differ significantly (*p* < 0.05). Lin and Quad show linear and quadratic effects of different dietary CEO levels.

As shown in Figure 3, higher *SOD1*, *GPX1*, and *Nrf2* mRNA expressions and a lower *Keap1* mRNA expression in the CEO60 group were observed compared to those in the control group (*p* < 0.05). Dietary CEO addition up-regulated *SOD2* mRNA expression relative to the control group (*p* < 0.05), and the expression of *SOD2* mRNA was higher in the CEO60 group than in the CEO40 and CEO80 groups (*p* < 0.05). Furthermore, the CEO60 and CEO80 groups had higher mRNA expressions of *CAT* than the control and CEO40 groups (*p* < 0.05). As dietary CEO addition increased, the mRNA expressions of *SOD2*, *CAT*, and *GPX1* increased (linear and quadratic, *p* < 0.05), and *Keap1* expression decreased (linear, *p* = 0.039; quadratic, *p* = 0.007); *SOD1* expression showed a quadratic change (*p* = 0.033).

### 3.8. Cecal Short-Chain Fatty Acid Concentrations

The impacts of dietary CEO addition on cecal SCFA concentrations in broilers are displayed in Table 6. Relative to the control group, a dietary supplementation with 60 mg/kg CEO increased acetic acid and isovaleric acid concentrations in the cecal contents compared with the control group (*p* < 0.05) and increased cecal propionic acid and total SCFA concentrations compared with the control, CEO40, and CEO80 groups (*p* < 0.05). The cecal propionic acid concentration was quadratically changed by increasing the CEO addition (*p* = 0.004). Moreover, the dietary inclusion of CEO elevated cecal butyric acid content (linear, *p* = 0.004; quadratic, *p* < 0.001), and the CEO addition groups had higher butyric acid contents than the control group (*p* < 0.05).

### 3.9. Cecal Bacterial Composition and Diversity

#### 3.9.1. Cecal Bacterial Alpha Diversity

**Figure 4:**
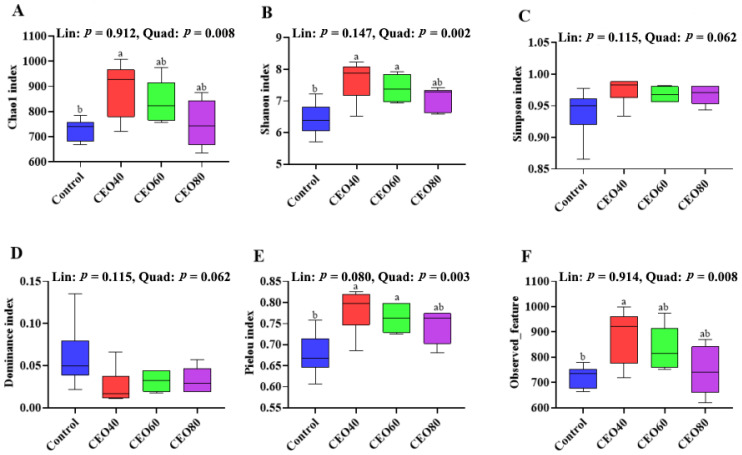
Effects of thymol–carvacrol cocrystals on cecal bacterial alpha diversity of broilers at 42 days of age. (**A**) Chao1 index; (**B**) Shannon index; (**C**) Simpson index; (**D**) Dominance index; (**E**) Pielou index; (**F**) Observed_feature. Control: basal diet without thymol–carvacrol cocrystal supplementation. CEO40, CEO60, and CEO80: basal diet supplemented with thymol–carvacrol cocrystals at 40, 60, and 80 mg/kg, respectively. Data in the bar chart are presented as mean ± SE. *n* = 6. a, b: means differ significantly (*p* < 0.05). Lin and Quad show linear and quadratic effects of different dietary CEO levels.

As shown in Figure 4, relative to the control group, the CEO40 group showed a higher Chao1 index (Figure 4A), Shannon index (Figure 4B), Pielou index (Figure 4E), and Observed_feature number (Figure 4F) (*p* < 0.05), and the CEO60 group exhibited a greater Shannon index and Pielou index (*p* < 0.05). The four indexes showed quadratic changes (*p* < 0.05) with increasing dietary CEO inclusion. Among the four groups, no significant differences were found in the Simpson (Figure 4C) and Dominance (Figure 4D) indexes of cecal microbiota (*p* > 0.05).

#### 3.9.2. Cecal Bacterial Beta Diversity

**Figure 5:**
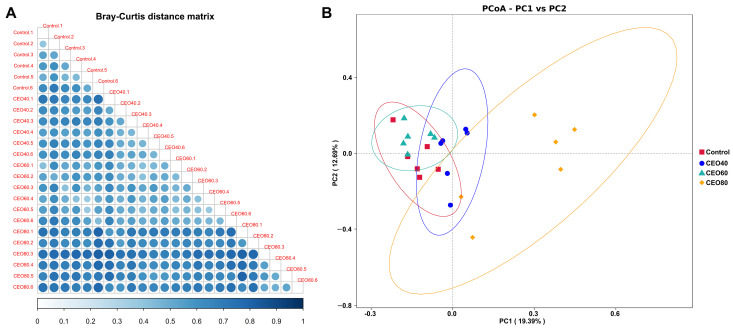
Effects of thymol–carvacrol cocrystals on cecal bacterial beta diversity of broilers at 42 days of age. (**A**) Triangular heatmap of Bray–Curtis distance matrix between samples; (**B**) Principal Co-ordinate Analysis (PCoA) based on Bray–Curtis distance. Control: basal diet without thymol–carvacrol cocrystal supplementation. CEO40, CEO60, and CEO80: basal diet supplemented with thymol–carvacrol cocrystals at 40, 60, and 80 mg/kg, respectively. *n* = 6.

The heatmap results (Figure 5A), generated using the Bray-Curtis distance matrix, illustrated that the greatest distance was observed between the control and CEO80 groups, whereas the least distance was found between the control and CEO60 groups. The PCoA profile based on the Bray-Curtis distance (Figure 5B) further showed a distinct separation between samples from different groups. Consistently, the ANOSIM (Supplemental Table S4) confirmed significant differences in the microbial community structure among the four groups (*p* < 0.05).

#### 3.9.3. Relative Abundance at the Phylum Level

**Figure 6:**
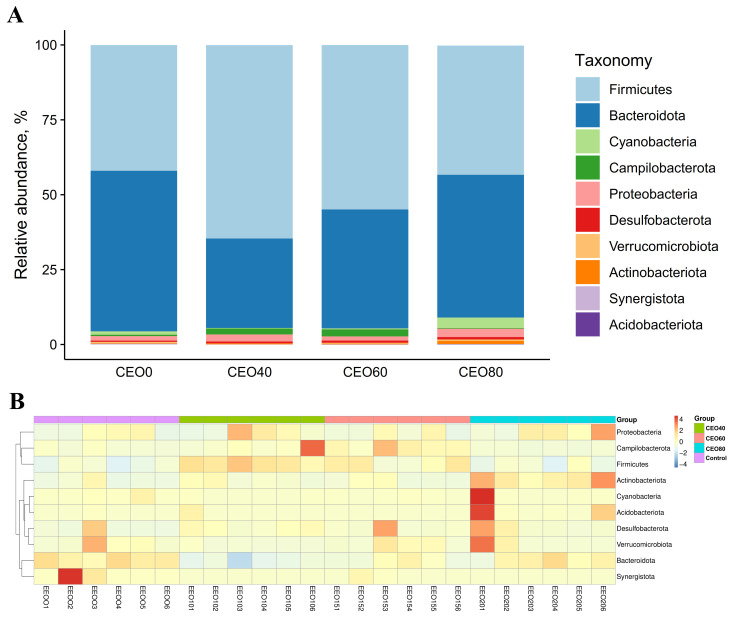
Effects of thymol–carvacrol cocrystals on the cecal bacterial composition and the relative abundance at the phylum level in broilers at 42 days of age. (**A**) Relative abundance of cecal bacteria at the phylum level; (**B**) heatmap of the top 10 phyla in broiler cecum. Control: basal diet without thymol–carvacrol cocrystal supplementation. CEO40, CEO60, and CEO80: basal diet supplemented with thymol–carvacrol cocrystals at 40, 60, and 80 mg/kg, respectively. *n* = 6.

The top ten phyla of cecal microbiota are shown in Figure 6A. Firmicutes and Bacteroidota emerged as the most predominant phyla across all groups, collectively accounting for over 90% of the total bacterial relative abundance. The heatmap (Figure 6B) exhibits the discrepancy in the relative abundance among all samples at the phylum level. As shown in Supplemental Figure S1, both the CEO40 group and the CEO60 group showed significantly greater relative abundances of Firmicutes and Campilobacterota than the control and CEO80 groups (*p* < 0.05). The Bacteroidota abundance was lower in the CEO40 and CEO 60 groups than in the control group (*p* < 0.05), and lower in the CEO40 group than in the CEO60 group (*p* < 0.05). The CEO80 group demonstrated a greater relative abundance of Cyanobacteria relative to both the CEO40 and CEO60 groups (*p* < 0.05), and of Actinobacteriota relative to the control and CEO60 groups (*p* < 0.05). Conversely, the CEO80 group exhibited a lower relative abundance of Synergistota than the control group (*p* < 0.05). In addition, the CEO40 group had a lower relative abundance of Verricomicrobiota than the control and CEO80 groups (*p* < 0.05). With dietary CEO addition increasing, the relative abundances of Firmicutes and Bacteroidota demonstrated quadratic changes (*p* < 0.001); the relative abundances of Actinobacteriota (linear, *p* = 0.003; quadratic, *p* < 0.001) and Synergistota (linear, *p* = 0.036) decreased.

#### 3.9.4. Relative Abundance at the Genus Level

**Figure 7:**
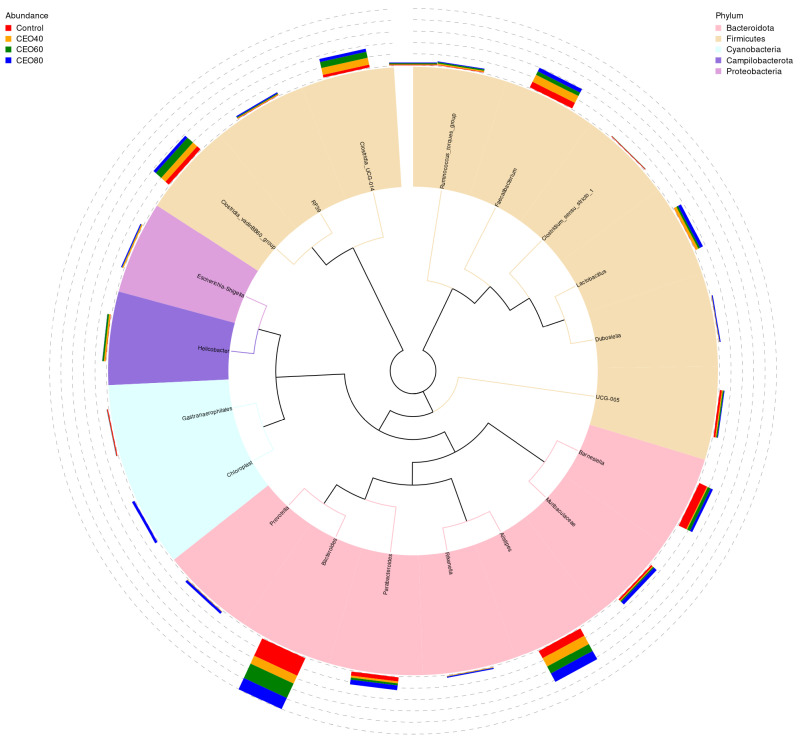
A phylogenetic tree constructed based on the top five most abundant genera in the cecal content of broilers at 42 days of age. The relative abundance of these genera is illustrated by the color variation in the branches and the corresponding fan-shaped representations. The stacked segments outside the fan shapes denote their proportional distribution across different treatments. Control: basal diet without thymol–carvacrol cocrystals supplementation. CEO40, CEO60, and CEO80: basal diet supplemented with thymol–carvacrol cocrystals at 40, 60, and 80 mg/kg, respectively. *n* = 6.

The top 20 genera with relative abundance are shown in Figure 7. In the control group, the dominant genera were Bacteroides, Barnesiella, Faecalibacterium, and Alistipes. In the CEO40 group, the dominant genera included Bacteroides, Alistipes, Faecalibacterium, Clostridia_vadinBB60_group, and Clostridia_UCG-014. The CEO60 group was dominated by Bacteroides, Alistipes, Clostridia_vadinBB60_group, and Clostridia_UCG-014, while Bacteroides and Alistipes were predominant in the CEO80 group. Compared to the control, Bacteroides and Barnesiella were less abundant in the CEO40 group, and Clostridia_vadinBB60_group was more abundant in the CEO60 group (*p* < 0.05; Supplemental Table S5). Clostridia_UCG-014 abundance increased in the CEO40 and CEO60 groups compared to the control and was higher in the CEO40 group than the CEO80 group (*p* < 0.05). CEO supplementation increased Lactobacillus and Escherichia-Shigella abundances (*p* < 0.05), with the latter being higher in the CEO40 and CEO80 groups. Supplementing with 60 mg/kg CEO increased Ruminococcus_torques_group abundance (*p* < 0.05). The CEO40 and CEO80 groups had lower Clostridium_sensu_stricto_1 abundances than the control and CEO60 groups (*p* < 0.05). CEO addition reduced the cecal Gastranaerophilales abundance compared to the control (*p* < 0.05), with CEO40 showing lower levels than CEO60 (*p* < 0.05). CEO60 increased Ruminococcus_torques_group abundance over the control (*p* < 0.05), and CEO80 had a higher Rikenella abundance than the other groups (*p* < 0.05). Increasing CEO raised Lactobacillus and Rikenella abundances and decreased Gastranaerophilales (linear and quadratic, *p* < 0.05), with quadratic changes in Barnesiella, Clostridia_vadinBB60_group, Clostridia_UCG-014, and Ruminococcus_torques_group (*p* < 0.05).

## 4. Discussion

It has been stated that feeding diets containing thymol and carvacrol have growth-promoting effects on animals [15,24]. In the current study, dietary CEO supplementation linearly and quadratically increased the BW, ADG, and ADFI of broilers. Particularly, the inclusion of 40 and 60 mg/kg CEO elevated the ADG and reduced the F/G during day 0 to 21, as well as elevating the BW at day 21. Since supplementing 40 and 60 mg/kg CEO to the diets did not affect the ADFI from day 0 to 21, the increase in ADG was likely related to the improvement of intestinal development and nutrient utilization during day 0 to 21. Consistently, Zhang et al. [25] also reported that the addition of oregano EO containing thymol and carvacrol increased the ADG and the feed conversion ratio of broilers during day 1 to 21. In this study, of particular note was that the inclusion of 60 mg/kg CEO significantly elevated the BW and the ADG and decreased the F/G throughout the 42 d trial. A previous study showed that dietary supplementation with 60, 100, and 200 mg/kg thymol and carvacrol linearly increased the ADG and feed efficiency of broilers in a 42 day study [6]. Ding et al. [7] also exhibited that supplementing 200, 400 and 600 mg/kg EOs containing carvacrol and thymol to broiler diets increased the ADG and BW from day 1 to 21, 22 to 42, and 1 to 48, and reduced the F/G from day 22 to 42 and 1 to 48, with the 400 mg/kg supplementation showing the most beneficial effects. Additionally, we found that the ADFIs of broilers during day 0 to 21 and 0 to 42 were increased by the 80 mg/kg CEO addition, but no significant difference was found in ADFI over day 22 to 42. Similarly, Ding et al. [7] demonstrated the increased ADFI of broilers during day 1 to 21 of EO (containing carvacrol and thymol) supplementation, but its effect of promoting appetite had disappeared during day 22 to 42. These findings align with the reported effects of thymol–carvacrol supplementation in weaned pigs [24], demonstrating consistent growth-promoting properties across species. However, Zhang et al. [25] indicated that the ADFI was not affected by the addition of 200 mg/kg oregano EOs containing 2.64% carvacrol and 1.3% thymol in broilers. The conflicting findings may depend on differences in the effective concentrations of carvacrol and thymol in the diets. Based on the above, 60 mg/kg CEO supplementation promoted the production performance of broilers in this study.

Serum biochemistry provides insights into the physiological and nutritional status. The TP, TG, GLU, and UREA serve as the key indicators of nutrient metabolism [26]. The TP, synthesized by the liver, can be used to reflect the protein nutritional status and the liver function of the body [27]. The serum activities of ALT and AST are the other two reliable biomarkers for monitoring the liver function, as both enzymes are released into circulation when liver damage occurs [26]. Blood UREA is an important indicator for evaluating the filtering function of the kidneys, and the UREA concentration in the blood may increase when the kidneys are damaged [28]. El-Sayed et al. [29] have demonstrated that thymol and carvacrol can prevent cisplatin-induced nephrotoxicity in rats. Combined with the increased TP concentration and decreased ALT, AST, and UREA concentrations in the serum in the CEO60 group, it demonstrated that 60 mg/kg CEO supplementation had a protective effect on the liver and kidney functions of the broilers in the present study. Moreover, the addition of 60 mg/kg CEO also reduced the serum TG concentration, but elevated the serum GLU concentration in the present study. Studies have shown that thymol and carvacrol exhibit anti-obesity and anti-diabetic properties [30,31]. GLU serves as a direct energy source, and its increase may be advantageous for the rapid growth of broilers [32]. One possible reason may be the enhancement of amylase activity, which can facilitate carbohydrate utilization. Additionally, studies demonstrated that carvacrol could up-regulate the expression of GLU transport-related genes, thereby promoting GLU uptake and utilization [33]. Thus, dietary 60 mg/kg CEO supplementation could regulate the nutritional status and play a protective role in the liver and kidney of broilers.

The intestinal absorptive function is closely associated with the intestinal morphology [15]. Serum DAO and D-lactate concentrations are widely recognized as biomarkers for evaluating intestinal mucosal injury. Damage to the intestinal mucosa can increase intestinal permeability, allowing the leakage of intracellular DAO and bacteria-derived D-lactate from the gut into systemic circulation [23]. In this study, increasing the CEO supplementation decreased the serum DAO and D-lactate concentrations. Supplementing 60 mg/kg CEO to the diet showed lower serum DAO and D-lactate concentrations than the control group, suggesting that the CEO60 group contributed to preserving intestinal integrity and supporting its fundamental functions. Consistently, Li et al. [11] found that dietary CEO supplementation up-regulated the mRNA expressions of tight junction proteins OCLN and ZO-1. Our results also showed that supplementation with 60 mg/kg CEO improved intestinal morphology, with increased VH and V/C ratios contributing to an expanded absorptive surface area, thereby enhancing the efficiency of nutrient uptake [34]. Consistently, Li et al. [11] indicated that additions of 30 and 60 mg/kg of CEO increased VH and the ratio of VH to CD in the jejunum of broilers. It is well known that the digestive physiology of broilers determines their nutrient absorption efficiency. A previous study demonstrated that oregano EO had positive effects on the activities of intestinal digestive enzymes, including trypsin, chymotrypsin, lipase, and amylase [25]. Similarly, in the CEO60 group, the enhanced activities of jejunal trypsin and amylase improved the digestibility of protein and starch, which in turn led to the observed increases in serum TP and GLU concentrations. In brief, dietary 60 mg/kg CEO supplementation could improve the intestinal morphology, maintain the integrity of the intestine, and enhance the activities of digestive enzymes.

The redox balance of broilers is closely associated with their growth performance, overall health status, and intestinal development [14]. As an important organ functioning in nutrient digestion and absorption, the intestine is immediately exposed to the oxidants in feeds, resulting in intestinal barrier damage. Moreover, the oxidants (such as the superoxide anion and H_2_O_2_) absorbed in the gut can enter the liver via the portal vein, inducing oxidative injury of the liver [35]. Both non-enzymatic and enzymatic antioxidant systems play important roles in protecting organisms from oxidative damage. GSH-Px, CAT, and SOD are the three endogenous antioxidant enzymes protecting cells against oxidative stress. SOD, as a first-line defender of the antioxidant system, transforms the superoxide anion radical (O^2−^) into hydrogen peroxide (H_2_O_2_), thereby reducing oxidative damage induced by ROS [36]. Both CAT and GSH-Px are important antioxidant enzymes that counteract oxidative stress by decomposing low-toxicity H_2_O_2_ into non-toxic H_2_O [36]. GSH is a key component of the non-enzyme antioxidant system, is the indispensable substance for the decomposition of H_2_O_2_ by various GSH-Pxs, and has a major role as a protector of biological structures and functions [37]. In the current study, dietary CEO supplementation linearly and/or quadratically boosted the antioxidant enzymes activities. Particularly, 60 mg/kg CEO enhanced the activities of SOD, CAT, and GSH in the serum, liver, and small intestine, as well as the activity of GSH-Px in the serum compared with the control group. The CEO60 group also showed lower MDA concentrations in the serum, liver, and small intestine. MDA is an end-product of polyunsaturated fatty acid peroxidation, serving as a reliable biomarker of oxidative stress, which can be mitigated through the activation of antioxidant enzymes [2]. Zhang et al. [25] also manifested that dietary 200 mg/kg oregano EO addition enhanced the antioxidant capacity by increasing the activities of GSH-Px and SOD and total antioxidant capacity in the serum. The enhanced antioxidant enzyme activities could be attributed to the up-regulated expressions of related genes in the liver and small intestine. In current study, the inclusion of 60 mg/kg CEO elevated Nrf2 expression and decreased Keap1 expression in the liver and small intestine. The Nrf2/Keap1 signaling pathway plays an important role in protecting cells against oxidative damage, and increasing the mRNA expression level of Nrf2 could enhance the activities of antioxidative enzymes [38]. Previous studies have demonstrated that both thymol and carvacrol can activate the Nrf2/Keap 1 pathway [39,40]. Overall, dietary 60 mg/kg CEO supplementation enhanced systematic, intestinal, and hepatic antioxidant capacity via the Nrf2/Keap1 signaling pathway.

The intestinal microbiota not only functions as a protective barrier, but also actively participates in physiological processes, including nutrient digestion and metabolic regulation [41,42]. In this study, dietary supplementations of 40 and 60 mg/kg showed higher alpha diversity indexes and obviously different microbiota community complexes compared with the control group. The Chao 1 index, Shannon index, Pielou index, and Observed_feature were often combined to estimate community diversity, richness, and evenness. Chronic intestinal oxidative stress and inflammation could result in the decreased alpha diversity [43]. Yang et al. [42] also showed that thymol and carvacrol complexes enhanced the microbial richness and diversity and reduced the diarrhea index in broilers. Furthermore, our study revealed that the CEO40 and CEO60 groups had an increased Firmicutes abundance and reduced Bacteroidota abundance compared with the control group. Firmicutes and Bacteroidota are the two dominant phyla in the broiler intestine [44]. The Firmicutes/Bacteroidota ratio has been widely regarded as a key indicator of broiler health. Previous studies also demonstrated that oregano oil supplementation increased the relative abundance of Firmicutes in yellow-feather broilers [45]. Numerous genera (such as Lactobacillus and Ruminococcus_torques_group) within Firmicutes could produce volatile fatty acids, which played a crucial role in nutrient absorption, energy metabolism, and growth performance [46]. Consistently, in this study, CEO supplementation increased butyric acid content, and the CEO60 group showed the highest acetic acid, propionic acid, butyric acid, isovalerate acid, and total SCFA concentrations in the cecum of broilers. In line with the findings, Clostridia_vadinBB60_group and Clostridia_UCG-014, which belong to the phylum Firmicutes, are positively associated with the production of butyric acid [46]. The SCFAs produced by the intestinal microbiota could inhibit intestinal oxidative stress [47,48]. Additionally, the enrichment of Clostridia_vadinBB60_group has been linked to improved growth performance, characterized by an increased BW gain and reduced F/G in broilers [49]. Another key genus, Lactobacillus, is widely recognized for its probiotic properties, including antioxidant, antiviral, and antibacterial effects [50]. Wu et al. [51] also indicated that Lactobacillus species played a crucial role in maintaining intestinal epithelial regeneration and homeostasis, while also exhibiting reparative effects on damaged intestinal tissue. Dietary oregano oil supplementation was shown to promote the proliferation of Lactobacillus in yellow-feather broilers [45]. Consistently, our study found that CEO supplementation increased Lactobacillus abundance in the broiler cecum, while 60 mg/kg CEO supplementation stimulated the growth of Clostridia_vadinBB60_group and Clostridia_UCG-014, suggesting a beneficial role of CEO in modulating intestinal microbiota to maintain intestinal health. In the present study, the reduction in Bacteroidota observed in the CEO40 and CEO60 groups may be attributed to decreased abundances of Bacteroides and Barnesiella, with the latter being recognized as a potential pathogen in broilers associated with inflammation [52]. Moreover, Bacteroides showed a negative correlation with the feed conversion ratio in a previous study [53]. Tiihone et al. [54] also found that dietary addition with a blend of EO containing 15 mg/kg thymol and 5 mg/kg cinnamaldehyde changed the cecal acetic acid, butyric acid, and isovaleric acid concentrations in broilers. A previous study in beef cattle demonstrated that dietary supplementation with oregano essential oil, rich in thymol and carvacrol, elevated the concentration of total SCFAs, particularly butyric acid [55]. Isovaleric acid, a branched SCFA, is primarily produced by the oxidative deamination or decarboxylation of leucine, isoleucine, and valine following protein degradation. It has been reported to improve growth performance and ameliorate intestinal inflammation in a mouse model of chronic restraint stress, an effect mediated through the inhibition of NF-κB activation [56]. The combined evidence of increased trypsin activity, elevated serum TP, and decreased serum UREA collectively suggests that the rise in isovaleric acid originated from the fermentation of endogenous proteins (e.g., shed epithelial cells) or balanced microbial metabolism in a healthier gut, rather than from an impaired dietary protein digestion. An enhanced protein digestion in the proximal gut could improve nutrient absorption, reducing the flow of undigested protein to the cecum and increasing the relative abundance of fermentable fibers for the microbiota. This shift in substrate composition might consequently promote broader microbial fermentation, resulting in elevated cecal SCFA levels. Above all, dietary CEO addition promoted the proliferation of Lactobacillus in the cecum of broilers. Moreover, the dietary inclusion of 40 and 60 mg/kg CEO could increase the richness and evenness of cecal microbiota, alter the microbiota community structures, and change the abundances of dominate bacteria in the cecum of broilers, which contributed to the increased concentrations of SCFAs. It might be another reason for the enhanced antioxidant capacity and improved intestinal development in the CEO60 group.

The superior benefits observed at 60 mg/kg compared to 80 mg/kg may be attributed to the biphasic nature of phenolic compounds like thymol and carvacrol. Llana-Ruiz-Cabello et al. have reported that higher concentrations of thymol, carvacrol, and their mixture caused oxidative stress [5]. This finding underscores the critical importance of optimizing the concentration of thymol, carvacrol, and their mixtures for their effective use as antioxidants. The recommendation for 60 mg/kg CEO, however, is specific to the conditions of this study, including the cage-rearing system and the particular 1:1 thymol–carvacrol cocrystal (25% effective content) that was evaluated. Subsequent studies are warranted to elucidate the optimal dietary supplementation level of CEO for broilers subjected to pathogenic infection. The objective is to delineate a dosage that effectively mitigates the impacts of clinical disease, thereby providing a scientific basis for its application as an effective strategy to bolster health and performance in challenging environments.

## 5. Conclusions

Our findings collectively indicated that the dietary inclusion of CEO (containing 25% thymol and carvacrol) exerts advantageous effects on production performance, antioxidant capacity, and the health and function of the liver and gut in broilers, suggesting its potential as a natural additive in poultry production. Notably, a dosage of 60 mg/kg CEO demonstrated the greatest effects.

## Figures and Tables

**Table 1:** Effects of thymol–carvacrol cocrystals on the growth performance of broilers.

Items	Treatment ^1^				SEM	*p*-Value ^2^		
	Control	CEO40	CEO60	CEO80		Trt	Lin	Quad
BW, g								
Day 0	44.93	45.04	45.00	45.10	0.03	0.201	0.067	0.189
Day 21	938.50 ^c^	945.50 ^b^	948.00 ^a^	944.00 ^b^	0.70	<0.001	0.001	<0.001
Day 42	2596.13 ^b^	2616.75 ^ab^	2637.50 ^a^	2620.88 ^ab^	4.09	0.002	0.007	<0.001
ADG, g								
Day 0–21	42.55 ^c^	42.88 ^b^	43.00 ^a^	42.81 ^b^	0.03	<0.001	0.001	<0.001
Day 22–42	78.93 ^b^	79.58 ^ab^	80.45 ^a^	79.85 ^ab^	0.18	0.016	0.020	0.011
Day 0–42	60.74 ^c^	61.23 ^b^	61.73 ^a^	61.33 ^ab^	0.09	<0.001	0.002	<0.001
ADFI, g								
Day 0–21	50.76 ^b^	50.76 ^b^	50.83 ^b^	51.48 ^a^	0.06	<0.001	<0.001	<0.001
Day 22–42	130.74	131.08	131.15	131.74	0.17	0.207	0.039	0.115
Day 0–42	90.81 ^b^	90.92 ^ab^	90.99 ^ab^	91.43 ^a^	0.09	0.048	0.010	0.022
F/G								
Day 0–21	1.20 ^a^	1.18 ^b^	1.18 ^b^	1.19 ^a^	0.002	<0.001	0.589	<0.001
Day 22–42	1.66 ^a^	1.65 ^ab^	1.63 ^b^	1.65 ^ab^	0.003	0.009	0.119	0.010
Day 0–42	1.50 ^a^	1.48 ^ab^	1.47 ^b^	1.49 ^a^	0.002	<0.001	0.265	<0.001
Survival rate, %	95.83 ^c^	97.60 ^ab^	98.36 ^a^	96.62 ^bc^	0.13	0.005	0.316	0.010
Dressing percentage	90.93 ^b^	91.34 ^ab^	91.92 ^a^	91.38 ^ab^	0.12	0.018	0.067	0.017

Abbreviations: BW = body weight; ADFI = average daily feed intake; ADG = average daily gain; F/G = feed-to-gain ratio. ^1^ Control: basal diet without thymol–carvacrol cocrystal supplementation. CEO40, CEO60, and CEO80: basal diet supplemented with thymol–carvacrol cocrystals at 40, 60, and 80 mg/kg, respectively. ^2^ Trt, Lin, and Quad show treatment, linear, and quadratic effects of different dietary CEO levels. a, b, c: means differ significantly (*p* < 0.05). *n* = 8.

**Table 2:** Effects of thymol–carvacrol cocrystals on the serum biochemistry of broilers at 42 days of age.

Items	Treatment ^1^				SEM	*p*-Value ^2^		
	Control	CEO40	CEO60	CEO80		Trt	Lin	Quad
TP, mol/L	37.18 ^b^	38.20 ^b^	44.78 ^a^	37.77 ^b^	0.98	0.009	0.351	0.070
TG, mmol/L	0.52 ^a^	0.48 ^ab^	0.44 ^b^	0.49 ^ab^	0.01	0.049	0.143	0.031
TC, mmol/L	2.97	2.97	2.87	2.83	0.07	0.866	0.409	0.710
GLU, mmol/L	12.30 ^b^	12.83 ^ab^	13.73 ^a^	12.98 ^ab^	0.18	0.031	0.067	0.032
UREA, mg/mL	1.72 ^a^	1.63 ^ab^	1.51 ^b^	1.55 ^ab^	0.14	0.031	0.010	0.015
HDL, mmol/L	1.64	1.90	2.06	1.89	0.07	0.158	0.131	0.077
LDL, mmol/L	0.56	0.53	0.52	0.56	0.02	0.715	0.977	0.501
ALT, U/L	14.28 ^a^	9.80 ^b^	7.00 ^c^	8.17 ^bc^	0.64	<0.001	<0.001	<0.001
AST, U/L	298.03 ^a^	265.82 ^ab^	255.53 ^b^	262.19 ^b^	5.42	0.016	0.011	0.005
ALP, U/L	3570.20	2981.60	2817.10	3101.70	127.26	0.184	0.173	0.083

Abbreviations: TP = total protein; TG = triacyl glyceride; TC = total cholesterol; GLU = glucose; UREA = urea nitrogen; HDL = high-density lipoprotein; LDL = low-density lipoprotein; ALT = alanine transaminase; AST = aspartate aminotransferase; ALP = alkaline phosphatase. ^1^ Control: basal diet without thymol–carvacrol cocrystal supplementation. CEO40, CEO60, and CEO80: basal diet supplemented with thymol–carvacrol cocrystals at 40, 60, and 80 mg/kg, respectively. ^2^ Trt, Lin, and Quad show treatment, linear, and quadratic effects of different dietary CEO levels. a, b, c: means differ significantly (*p* < 0.05). *n* = 8.

**Table 3:** Effects of thymol–carvacrol cocrystals on serum diamine oxidase (DAO) and D-lactate concentrations in broilers at 42 days of age.

Items	Treatment ^1^				SEM	*p*-Value ^2^		
	Control	CEO40	CEO60	CEO80		Trt	Lin	Quad
DAO, U/mL	9.25 ^a^	7.97 ^a^	5.83 ^b^	8.07 ^a^	0.31	<0.001	0.003	<0.001
D-lactate, μg/L	487.66 ^a^	483.63 ^a^	384.63 ^b^	395.61 ^b^	10.74	<0.001	<0.001	<0.001

^1^ Control: basal diet without thymol–carvacrol cocrystal supplementation. CEO40, CEO60, and CEO80: basal diet supplemented with thymol–carvacrol cocrystals at 40, 60, and 80 mg/kg, respectively. ^2^ Trt, Lin, and Quad show treatment, linear, and quadratic effects of different dietary CEO levels. a, b: means differ significantly (*p* < 0.05). *n* = 8.

**Table 4:** Effects of thymol–carvacrol cocrystals on antioxidant parameters in the serum, liver, and small intestine of broilers at 42 days of age.

Items	Treatment ^1^				SEM	*p*-Value ^2^		
	Control	CEO40	CEO60	CEO80		Trt	Lin	Quad
Serum								
SOD, U/mL	24.27 ^c^	34.23 ^b^	42.74 ^a^	35.51 ^b^	1.65	<0.001	<0.001	<0.001
CAT, U/mL	23.70 ^b^	34.15 ^ab^	41.45 ^a^	39.31 ^a^	2.25	0.014	0.004	0.005
GSH-Px, U/mL	507.03 ^b^	593.02 ^ab^	665.24 ^a^	607.92 ^ab^	17.51	0.006	0.013	0.003
GSH, μmol/L	8.61 ^b^	9.73 ^ab^	10.80 ^a^	9.97 ^ab^	0.27	0.024	0.028	0.012
MDA, nmol/mL	4.44 ^a^	3.31 ^b^	2.80 ^b^	3.06 ^b^	0.16	<0.001	<0.001	<0.001
Liver								
SOD, U/mg prot	30.10 ^b^	30.65 ^b^	32.15 ^a^	30.72 ^b^	0.18	<0.001	0.030	<0.001
CAT, U/mg prot	8.10 ^b^	9.96 ^b^	15.05 ^a^	11.67 ^ab^	0.79	0.007	0.023	0.014
GSH-Px, U/mg prot	18.54	20.64	20.63	19.90	1.61	0.969	0.785	0.789
GSH, μmol/g prot	11.64 ^b^	14.25 ^ab^	19.45 ^a^	13.65 ^ab^	1.02	0.032	0.224	0.047
MDA, nmol/mg prot	1.11 ^a^	0.95 ^ab^	0.84 ^b^	0.93 ^ab^	0.03	0.012	0.018	0.005
Small intestine								
SOD, U/mg prot	52.85 ^c^	58.46 ^bc^	71.03 ^a^	67.34 ^ab^	1.85	<0.001	<0.001	<0.001
CAT, U/mg prot	17.80 ^b^	22.00 ^ab^	30.75 ^a^	23.63 ^ab^	1.52	0.013	0.052	0.020
GSH-Px, U/mg prot	368.61	400.11	392.96	381.01	18.92	0.948	0.864	0.847
GSH, μmol/g prot	90.91 ^b^	97.70 ^b^	142.14 ^a^	112.05 ^ab^	6.55	0.017	0.064	0.062
MDA, nmol/mg prot	2.06 ^a^	1.47 ^ab^	1.01 ^b^	1.40 ^ab^	0.11	0.005	0.014	0.002

Abbreviations: SOD = superoxide dismutase; CAT = catalase; GSH-Px = glutathione peroxidase; GSH = glutathione; MDA = malondialdehyde. ^1^ Control: basal diet without thymol–carvacrol cocrystals supplementation. CEO40, CEO60, and CEO80: basal diet supplemented with thymol–carvacrol cocrystals at 40, 60, and 80 mg/kg, respectively. ^2^ Trt, Lin, and Quad show treatment, linear, and quadratic effects of different dietary CEO levels. a, b, c: means differ significantly (*p* < 0.05). *n* = 8.

**Table 5:** Effects of thymol–carvacrol cocrystals on the jejunal digestive enzyme activity of broilers at 42 days of age.

Items, U/gprot	Treatment ^1^				SEM	*p*-Value ^2^		
	Control	CEO40	CEO60	CEO80		Trt	Lin	Quad
Trypsin	204.42 ^b^	225.38 ^ab^	245.73 ^a^	224.71 ^ab^	4.02	<0.001	0.020	<0.001
Lipase	43.61	46.88	49.28	49.17	0.99	0.135	0.027	0.059
Amylase	242.73 ^b^	257.43 ^ab^	267.91 ^a^	253.32 ^ab^	3.31	0.046	0.157	0.023

^1^ Control: basal diet without thymol–carvacrol cocrystal supplementation. CEO40, CEO60, and CEO80: basal diet supplemented with thymol–carvacrol cocrystals at 40, 60, and 80 mg/kg, respectively. ^2^ Trt, Lin, and Quad show treatment, linear, and quadratic effects of different dietary CEO levels. a, b: means differ significantly (*p* < 0.05). *n* = 8.

**Table 6:** Effects of thymol–carvacrol cocrystals on volatile fatty acid contents in cecum of broilers at 42 days of age.

Items, ug/g	Treatment ^1^				SEM	*p*-Value ^3^		
	Control	CEO40	CEO60	CEO80		Trt	Lin	Quad
Acetic acid	145.25 ^b^	161.82 ^ab^	187.73 ^a^	153.35 ^ab^	5.85	0.046	0.349	0.052
Propionic acid	34.97 ^b^	39.54 ^b^	51.44 ^a^	40.46 ^b^	1.65	<0.001	0.052	0.004
Isobutyric acid	9.73	12.30	11.20	9.69	0.65	0.441	0.841	0.297
Butyric acid	42.21 ^b^	63.16 ^a^	77.59 ^a^	66.00 ^a^	3.54	<0.001	0.004	<0.001
Valeric acid	5.51	5.69	7.68	6.01	0.55	0.511	0.491	0.567
Isovaleric acid	4.39 ^b^	5.62 ^ab^	8.00 ^a^	4.53 ^ab^	0.512	0.036	0.555	0.055
Caproate acid	0.11	0.16	0.11	0.12	0.01	0.285	0.929	0.616
Total SCFAs ^2^	242.16 ^b^	288.29 ^b^	343.75 ^a^	280.16 ^b^	9.45	<0.001	0.349	0.052

^1^ Control: basal diet without thymol–carvacrol cocrystals supplementation. CEO40, CEO60, and CEO80: basal diet supplemented with thymol–carvacrol cocrystals at 40, 60, and 80 mg/kg, respectively. ^2^ Total SCFAs were defined as the sum of acetic, propionic, butyric, isobutyric, valeric, isovaleric, and caproic acids. ^3^ Trt, Lin, and Quad show treatment, linear, and quadratic effects of different dietary CEO levels. a, b: means differ significantly (*p* < 0.05). *n* = 8.

## Data Availability

All sequencing data have been deposited in the NCBI Sequence Read Archive under accession number PRJNA1215442 (Illumina sequences).

## Supplementary Materials

The following supporting information can be downloaded at https://www.mdpi.com/article/10.3390/antiox14111323/s1. Figure S1: Effects of thymol–carvacrol cocrystals on the relative abundance of cecal microbiota at the phylum level in broilers at 42 days of age; Table S1: Ingredient composition and nutrient levels of basal diets (as-fed basis); Table S2: PCR primer gene sequences; Table S3: Effects of thymol–carvacrol cocrystals on slaughter performance of broilers at 42 days of age; Table S4: ANOSIM analysis for beta diversity of cecal microbiota of broilers at 42 days of age; Table S5: Effects of thymol–carvacrol cocrystals on the relative abundance of cecal microbiota of broilers at 42 days of age at the genus level.

## Abbreviations

The following abbreviations are used in this manuscript:

ROS	Reactive oxygen species
EOs	Essential oils
BW	Body weight
CEO	Thymol–carvacrol cocrystals
ADFI	Average daily feed intake
ADG	Average daily gain
F/G	The ratio of feed to gain
TP	Total protein
TG	Triacylglycerol
TC	Cholesterol
GLU	Glucose
UREA	Urea nitrogen
HDL	High-density lipoprotein
LDL	Low-density lipoprotein
ALP	Alkaline phosphatase
AST	Aspartate transaminase
ALT	Alanine transaminase
SOD	Superoxide dismutase
CAT	Catalase
GSH-Px	Glutathione peroxidase
GSH	Glutathione
MDA	Malondialdehyde
DAO	Diamine oxidase
CD	Crypt depth
VH	Villus height
V/C	VH to CD ratio
SCFA	Short-chain fatty acid
PCoA	Principal Coordinate Analysis
*SOD1*	Superoxide dismutase 1
*SOD2*	Superoxide dismutase 2
*GPX1*	Glutathione peroxidase 1
*Keap1*	Kelch-like ECH-associated protein 1
*Nrf2*	Nuclear factor erythroid 2-related factor 2
