# Supplementary material for: Dietary Thymol–Carvacrol Cocrystal Supplementation Improves Growth Performance, Antioxidant Status, and Intestinal Health in Broiler Chickens
Source: Antioxidants (Basel). 2025 Nov 1;14(11):1323. doi: 10.3390/antiox14111323 (PMC12649561; doi:10.3390/antiox14111323)
Supplement: Supplementary file 1 [file antioxidants-14-01323-s001.zip › antioxidants-3919732-supplementary.pdf]

Table S1. Ingredients composition and nutrient levels of basal diets (as-fed basis).

Ingredients, %	1-14 d	15-28 d	29-42 d
Corn	36.62	39.53	42.10
Soybean meal, 46% CP	25.70	23.90	22.10
Coarse rice	15.00	12.50	10.00
Wheat flour	8.00	8.00	8.00
Corn gluten meal	2.00	2.00	2.00
Cottonseed meal	4.00	4.00	4.00
Hydrolyzed feather meal	1.50	1.50	1.50
$CaHPO_4$	1.77	1.45	1.18
Pulverized Limestone	0.87	0.60	0.54
Duck fat	1.50	3.70	5.90
Choline chloride, 50%	0.20	0.20	0.20
L-lysine HCl, 98%	0.40	0.29	0.23
DL-methionine, 98%	0.32	0.27	0.23
L-tryptophan, 98%	0.12	0.06	0.02
Premix[1]	2.00	2.00	2.00
Total	100.00	100.00	100.00
Nutrient contents, % [2]			
Metabolic energy, MJ/kg	12.58	13.15	13.68
Crude protein	21.52	20.51	19.54
Ether extract	3.82	6.00	8.16
Crude fiber	2.56	2.47	2.37
Calcium	0.95	0.75	0.64
Available phosphorus	0.50	0.42	0.36
Lysine	1.32	1.18	1.08
Methionine + Cystine	1.00	0.92	0.86
Threonine	0.88	0.79	0.72
Tryptophan	0.23	0.22	0.21

[1] The premix provided for per kg of basal diet: vitamin B_1, 2.40 mg; vitamin A, 9 600 IU; vitamin B_{12}, 0.01 mg; vitamin E, 24 IU; vitamin D_3 1 200 IU; vitamin K_3, 0.60 mg; vitamin B_2, 9.60 mg; niacin, 42.00 mg; pantothenic acid, 12.00 mg; folic acid, 0.66 mg; biotin, 0.21 mg; Mn ($MnSO_4 \cdot H_2O$), 120 mg; Cu ($CuSO_4 \cdot 5H_2O$), 8.00 mg; Zn ($ZnSO_4 \cdot H_2O$), 100 mg; Fe ($FeSO_4 \cdot H_2O$), 100 mg; I (KIO_3), 0.70 mg; Se (Na_2SeO_3), 0.30 mg. [2] The content of crude protein, ether extract, and calcium were measured, and others were calculated.

Table S2. PCR primer gene sequences.

Target gene	Primer sequence (5' to 3')	Product size, bp	Accession No
β-actin	F:5'-ATTGTCCACCGCAAATGCTTC-3'<br>R: 5'-AAATAAAGCCATGCCAATCTCGTC-3'	113	NM_205518.1
Keap1	F:5'-GCATCACAGCAGCGTGGAGAG-3'<br>R:5'-GCGTACAGCAGTCGGTTCAGC-3'	108	XM_025145847.1
SOD1	F:5'-CGCAGGTGCTCACTTCAATCC-3'<br>R:5'-CAGTCACATTGCCGAGGTCAC-3'	89	NM_205064.2
SOD2	F:5'-GCTGTATCAGTTGGTGTTCAAGGA-3'<br>R:5'-GCAATGGAATGAGACCTGTTGTTC-3'	130	NM_204211.2
CAT	F:5'-GGAGGTAGAACAGATGGCGTATG-3'<br>R:5'-CGATGTCTATGCGTGTCAGGAT-3'	114	NM_001031215.2
GPX1	F:5'-CGGCTTCAAACCCAACTTCAC-3'<br>R:5'-CTCTCTCAGGAAGGCGAACAG-3'	85	NM_001277853.3
Nrf2	F:5'-TCGCAGAGCACAGATACTTCAA-3'<br>R:5'-CTGGAGAAGCCTCATTGTCATCTA-3'	109	XM_013984303.2

Abbreviations: F = forward; R = reverse; *Keap1* = kelch-like ECH-associated protein 1; *SOD1* = superoxide dismutase 1; *SOD2* = superoxide dismutase 2; *CAT* = catalase; *GPX1* = glutathione peroxidase 1; *Nrf2* = nuclear factor erythroid 2-related factor 2.

Table 3. Effects of thymol-carvacrol cocrystals on slaughter performance of broilers at 42 days of age.

Items, %	Treatment[1]				SEM	*p*-value[2]		
	Control	CEO40	CEO60	CEO80		Trt	Lin	Quad
Percentage of semi-eviscerated yield	83.64	84.96	85.64	84.88	0.37	0.290	0.188	0.150
Percentage of full eviscerated yield	71.60	72.63	73.79	72.90	0.43	0.361	0.192	0.230
Percentage of abdominal fat	1.72	1.62	1.46	1.47	0.06	0.281	0.063	0.161
Percentage of breast muscle	22.99	23.31	24.23	22.74	0.41	0.629	0.970	0.566
Percentage of leg muscle	16.97	16.64	17.62	17.14	0.36	0.826	0.652	0.901

[1] Control, basal diet without thymol-carvacrol cocrystals supplementation. CEO40, CEO60, and CEO80, basal diet supplemented with thymol-carvacrol cocrystals at 40, 60, and 80 mg/kg, respectively. [2] Trt, Lin, and Quad showed treatment, linear, and quadratic effects of different dietary CEO levels. a,b: means differ significantly ($p < 0.05$). $n = 8$.

Table S4. ANOSIM analysis for beta diversity of cecal microbiota of broilers at 42 days of age.

Group-pair	*R* value	*p*-value
Control *vs.* CEO40	0.697	0.005
Control *vs.* CEO60	0.314	0.010
Control *vs.* CEO80	0.613	0.010
CEO40 *vs.* CEO60	0.372	0.010
CEO40 *vs.* CEO80	0.476	0.005
CEO60 *vs.* CEO80	0.581	0.010

Control, basal diet without thymol-carvacrol cocrystals supplementation. CEO40, CEO60, and CEO80, basal diet supplemented with thymol-carvacrol cocrystals at 40, 60, and 80 mg/kg, respectively. $n = 6$.

Table S5. Effects of thymol-carvacrol cocrystals on the relative abundance of cecal microbiota of broilers at 42 days of age at the genus level.

Items, %	Treatment[1]				SEM	*p*-value[2]		
	Control	CEO40	CEO60	CEO80		Trt	Lin	Quad
Bacteroides	24.32[a]	10.96[b]	20.05[ab]	15.73[ab]	1.86	0.029	0.181	0.154
Barnesiella	10.60[a]	1.21[b]	3.93[ab]	3.18[ab]	1.36	0.011	0.050	0.044
Clostridia_vadin BB60_group	5.62[bc]	7.18[ab]	9.20[a]	3.88[c]	0.56	<0.001	0.826	0.013
Clostridia_UCG-014	3.49[c]	8.89[a]	7.71[ab]	3.95[bc]	0.74	0.004	0.561	0.003
Lactobacillus	0.35[b]	3.38[a]	2.34[a]	4.20[a]	0.49	<0.001	0.006	0.024
Escherichia-Shigella	0.11[b]	1.47[a]	0.40[ab]	1.23[a]	0.24	0.006	0.203	0.352
Clostridium_sensu_ stricto_1	0.88[a]	0.04[b]	0.32[a]	0.04[b]	0.18	<0.001	0.117	0.236
Gastranaerophilales	1.13[a]	0.10[c]	0.34[b]	0.15[bc]	0.16	<0.001	0.024	0.038
Ruminococcus_ torques_group	0.70[b]	1.48[ab]	1.71[a]	0.96[ab]	0.16	0.014	0.292	0.049
Rikenella	0.24[b]	0.50[b]	0.36[b]	1.32[a]	0.14	<0.001	0.014	0.014

[1] Control, basal diet without thymol-carvacrol cocrystals supplementation. CEO40, CEO60, and CEO80, basal diet supplemented with thymol-carvacrol cocrystals at 40, 60, and 80 mg/kg, respectively. [2] Trt, Lin, and Quad showed treatment, linear, and quadratic effects of different dietary CEO levels. a, b, c: means differ significantly ($p < 0.05$). $n = 6$.

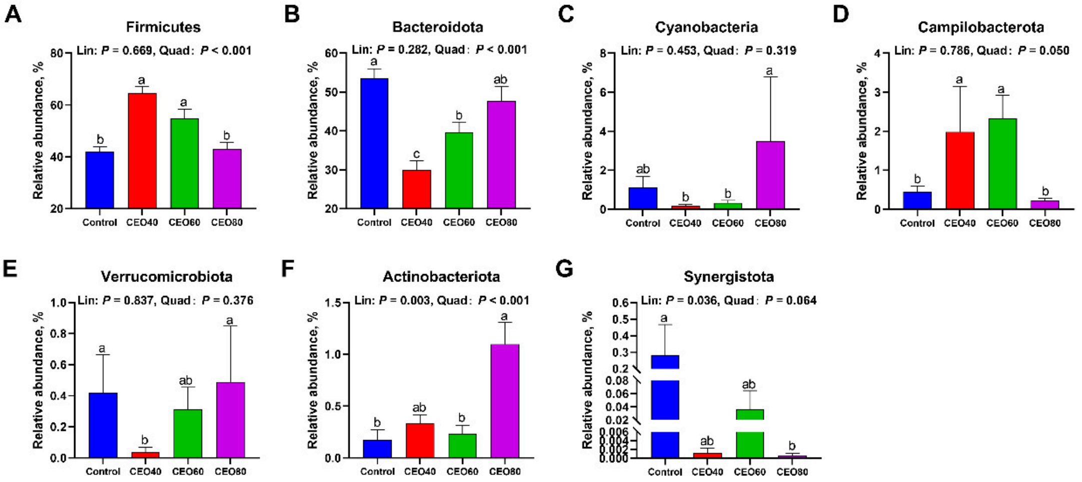

Figure S1. Effects of thymol-carvacrol cocrystals on the relative abundance of cecal microbiota at the phylum level in broilers at 42 days of age. (A) Firmicutes; (B) Bacteroidota; (C) Cyanobacteria; (D) Campilobacterota; (E) Verrucomicrobiota; (F) Actinobacteria; (I) Synergistota. Control, basal diet without thymol-carvacrol cocrystals supplementation. CEO40, CEO60, and CEO80, basal diet supplemented with thymol-carvacrol cocrystals at 40, 60, and 80 mg/kg, respectively. Data in the bar chart are presented as mean ± SE. n = 6. $^{a, b, c}$ means differ significantly ($p < 0.05$). Lin and Quad showed linear and quadratic effects of different dietary CEO levels.
